# Association of tear fluid amyloid and tau levels with disease severity and neurodegeneration

**DOI:** 10.1038/s41598-021-01993-x

**Published:** 2021-11-22

**Authors:** Marlies Gijs, Inez H. G. B. Ramakers, Pieter Jelle Visser, Frans R. J. Verhey, Marjo P. H. van de Waarenburg, Casper G. Schalkwijk, Rudy M. M. A. Nuijts, Carroll A. B. Webers

**Affiliations:** 1 University Eye Clinic Maastricht, Maastricht University Medical Center (MUMC+), School for Mental Health and Neuroscience, Maastricht University, P. Debyelaan 25, 6229 HX Maastricht, The Netherlands; 2 Department of Psychiatry and Neuropsychology, Alzheimer Center Limburg, School for Mental Health and Neuroscience, Maastricht University, Maastricht, The Netherlands; 3 Department of Neurology, Alzheimer Center Amsterdam, Vrije Universiteit Medical Center, Neuroscience Campus Amsterdam, Amsterdam, The Netherlands; 4 Department of Internal Medicine, School for Cardiovascular Diseases (CARIM), Maastricht University Medical Center, Maastricht, The Netherlands

**Keywords:** Dementia, Neurochemistry, Diagnostic markers

## Abstract

There has been increasing interest in finding non-invasive biomarkers for neurodegenerative diseases such as Alzheimer’s disease (AD). This observational study investigated AD-specific biomarkers in tear fluid. Tear fluid was collected from a total of 65 subjects, including 23 patients with subjective cognitive decline (SCD), 22 patients with mild cognitive impairment (MCI), 11 dementia patients and 9 healthy controls (HC). Levels of amyloid-beta peptides (AB38, AB40, AB42), total-tau (t-tau) and phosphorylated-tau (p-tau) were determined using multiplex immunoassays. Levels of AB40 and t-tau were detectable in the vast majority (> 94%) of tear fluid samples. Cerebrospinal fluid (CSF) was available from a subset of patients. In this group, tear t-tau levels were significantly higher in people with dementia compared to SCD patients. Tear t-tau levels were elevated in patients with neurodegeneration (classified according to the A/T/N system) compared to patients without neurodegeneration. Negative correlations were found between CSF AB42 and CSF t-tau, and between CSF AB42 and tear t-tau. In summary, this study shows the potential of tau proteins in tear fluid to be associated with disease severity and neurodegeneration.

## Introduction

Aggregation of amyloid-beta (AB) peptide into extracellular plaques together with the presence of neurofibrillary tangles (NTS) of truncated and phosphorylated forms of the microtubule-stabilizing protein tau are central in the molecular pathogenesis of Alzheimer’s disease (AD)^1^. AB plaques and NTS can be visualized by cortical amyloid and tau PET ligand binding or postmortem immunohistochemistry^2,3^. These key pathological hallmarks are however not restricted to brain tissue. The retina, that is embryologically derived from the neural tube as a protrusion from the brain, shares many similarities with brain tissue. As such, AB plaques and tau accumulation have also been observed in postmortem retina samples of AD patients^4,5^. In addition, non-invasive retinal imaging was able to detect amyloid plaques in vivo^4^. The retina of AD patients is also characterized by widespread retinal ganglion cell losses, retinal nerve fiber layer (RNFL) thinning and optic nerve degeneration^6–10^.

In addition to its presence in AB plaques and NTS, AB and tau are also found as soluble forms in cerebrospinal fluid (CSF)^11–13^. Low CSF AB42 and elevated CSF phosphorylated tau (p-tau) and total tau (t-tau) are validated diagnostic biomarkers for AB plaques, NTS and neurodegeneration, respectively^3,14^. Other, more accessible, body fluids such as blood are known to contain AB and tau^12,14–16^. However, quantification of AD biomarkers in peripheral fluids is challenging, even with very sensitive techniques, because of their low abundance in these biological samples^17^. Because of its close proximity to the brain, there has been increasing interest in testing ocular fluids such as tear fluid, aqueous humor and vitreous body. Previous studies in cognitively healthy persons found soluble AB42 in aqueous humor^18^ and AB40, AB42 and neurofilament light (NfL) in vitreous humor^19,20^. Another study showed presence of the full-length amyloid-beta precursor protein (APP) in human tear fluid and the lacrimal gland (extraocular gland that produces tear fluid) of healthy persons^21^. However, it is not clear yet whether other AB species and tau proteins are present in tear fluid, and if they are, whether their concentrations differ between patients and controls.

Tear fluid is non-invasively collected via Schirmer’s strips, a paper strip which is gently placed in the lower eyelid where it absorbs tear fluid. This method, which is used as a standard test in clinical practice, is a simple and quick procedure to collect basal tears and is becoming increasingly relevant. Tear fluid (when extracted from paper strips,) can be used directly in (immuno-)assays.

In the present work, we explored whether amyloid-beta peptides (AB38, AB40 and AB42), t-tau and p-tau are present in tear fluid and if differences can be found between patients with cognitive impairment and healthy controls.

## Results

### Participants

The total study group consisted of 56 patients and nine cognitively healthy controls. Twenty-three patients received a diagnosis of SCD, 22 patients had a diagnosis of MCI and 11 patients had a clinical diagnosis of dementia (Table 1). Dementia patients and MCI patients were older than SCD patients, and dementia patients were older than healthy controls. Sex was comparable across diagnostic groups. Mini-Mental State Examination (MMSE) score of people with dementia and MCI was lower than that of SCD patients.

**Table 1:** Demographic and clinical characteristics and tear biomarkers of the total group.

	HC (n = 9)	SCD (n = 23)	MCI (n = 22)	Dementia (n = 11)
Age (years)	60 (16)	61 (13)	70 (13.75)^a^	74 (3)^b,c^
Male/female	4/5	14/9	12/10	6/5
MMSE	–	29.0 (2.00)	27.5 (2.75)^a^	24.5 (6.50)^c^
Tear AB38 (pg/mL)	59.10 (6.86)	636.60 (4163.70)	607.80 (1268.60)	1682.00 (3165.88)
Tear AB40 (pg/mL)	13.93 (8.58)	24.13 (26.76)	23.72 (29.71)	28.26 (34.12)
Tear AB42 (pg/mL)	4.61 (1.58)	75.18 (139.48)	17.37 (45.66)	29.90 (41.97)
Tear t-tau (ng/mL)	1.28 (2.14)	2.52 (2.38)	2.90 (3.88)	2.42 (6.64)
Tear p-tau (U/mL)	u.d	18.99 (15.92)	54.46 (51.55)	25.10 (33.18)

Data are shown as median (interquartile range).

*u.d* undetectable, *MMSE* Mini-Mental State Examination.

^a^p < 0.01 SCD vs MCI, ^b^p < 0.05 Dementia vs HC, ^c^p < 0.001 Dementia vs SCD, p-values are from the Kruskal–Wallis test.

#### Tear fluid biomarker detectability

Tear fluid was collected from both eyes of all 65 participants. Table 2 displays the number of samples with detectable levels of the three amyloid-beta peptides (38, 40 and 42), total-tau and p-tau. In the total study group, AB40 and t-tau were detectable in more than 94% of tear fluid samples, while AB38, AB42 and p-tau were detectable in less than 23% of tear fluid samples. Interestingly, tear p-tau was not detectable in tear samples from the HC group. In addition, AB42 was better detectable in HCs (78%) compared to patients (< 18%).

**Table 2:** Detectability.

Tear fluid biomarker detectability n (%)	HC (n = 9)	SCD (n = 23)	MCI (n = 22)	Dem (n = 11)	Total study group (n = 65)
Tear AB38	2 (22%)	3 (13%)	5 (22%)	2 (18%)	12 (18%)
Tear AB40	9 (100%)	23 (100%)	20 (87%)	11 (100%)	63 (97%)
Tear AB42	7 (78%)	2 (9%)	4 (17%)	2 (18%)	15 (23%)
Tear t-tau	9 (100%)	19 (83%)	22 (100%)	11 (100%)	61 (94%)
Tear p-tau	0 (0%)	4 (17%)	5 (22%)	3 (27%)	12 (18%)

Number (n) and percentage (%) of tear fluid samples with biomarkers above the LLOD.

#### Biomarker levels in the total study group

Table 1 shows tear fluid biomarker levels across diagnostic groups in the total study group. Tear fluid levels of AB38, AB40, AB42 and t-tau were higher in patients compared to HC, but differences were not statistically significant. Tear fluid levels of p-tau did not differ between patient groups.

#### Biomarker levels in the CSF group

**Figure 1:**
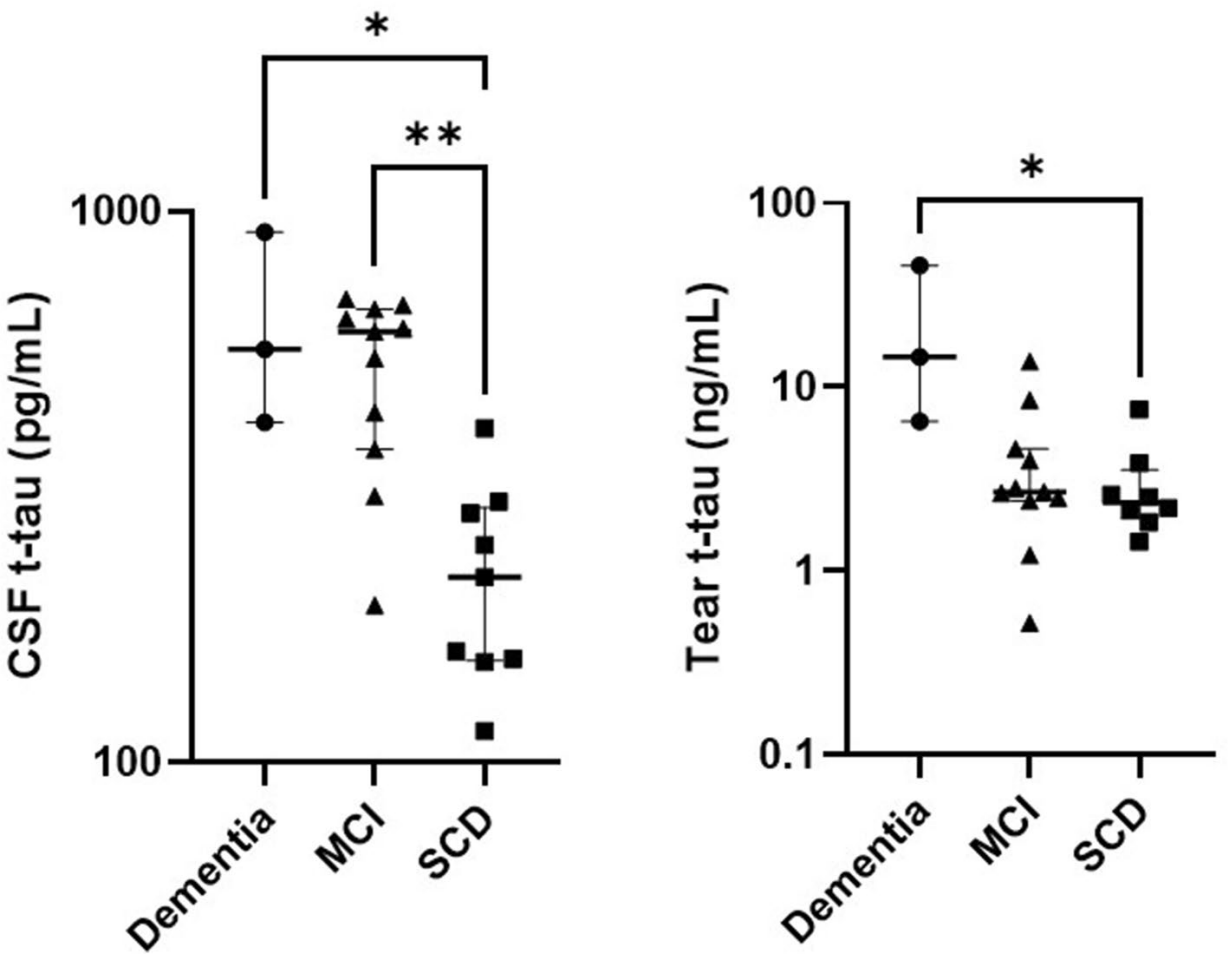
T-tau biomarker levels across diagnostic groups. Evaluation of t-tau in CSF and tear fluid of the CSF group. Bars represent median and IQR. P-values were determined using Kruskal–Wallis testing including Bonferroni correction. *p < 0.05, **p < 0.01.

In the CSF group, CSF was available for clinical diagnostic purposes from 23 patients (9 SCD patients, 11 MCI patients and 3 patients with dementia) (Table 3). Although CSF AB42 levels in dementia patients were lower than for MCI patients and SCD patients, these differences were not statistically significant. CSF t-tau levels were significantly increased in people with dementia and MCI patients compared to SCD patients (Fig. 1). The same trend was observed for CSF p-tau levels. Tear t-tau levels were significantly higher in people with dementia compared to SCD patients (Fig. 1).

**Table 3:** Demographic and clinical characteristics and tear biomarkers of the CSF group.

	SCD (n = 9)	MCI (n = 11)	Dem (n = 3)
Age (years)	60 (10)^a^	69 (8)	74 (6)
Male/female	5/4	7/4	2/1
MMSE	29 (2)	27 (4)	25 (11)
CSF AB42 (pg/mL)	1162 (345)	747 (468)	707 (512)
CSF t-tau (pg/mL)	217 (137)^b^	604 (294)^c^	562 (502)
CSF p-tau (pg/mL)	41 (21)^b^	77 (27)^c^	84 (52)
Tear AB40 (pg/mL)	21.61 (47.05)	23.33 (20.51)	77.47 (68.87)
Tear t-tau (ng/mL)	2.35 (1.62)^b^	2.69 (2.19)	14.54 (39.38)

Data are shown as median (interquartile range).

*MMSE* Mini-Mental State Examination, *CSF* cerebrospinal fluid.

^a^p < 0.01 Dementia vs SCD, ^b^p < 0.05 Dementia vs SCD, ^c^p < 0.01 MCI vs SCD, p-values are from the Kruskal–Wallis test or Fisher exact test, Tear AB38, tear AB42 and tear p-tau were excluded due to n < 3 in the CSF group.

#### Biomarker levels along the ATN classification

**Figure 2:**
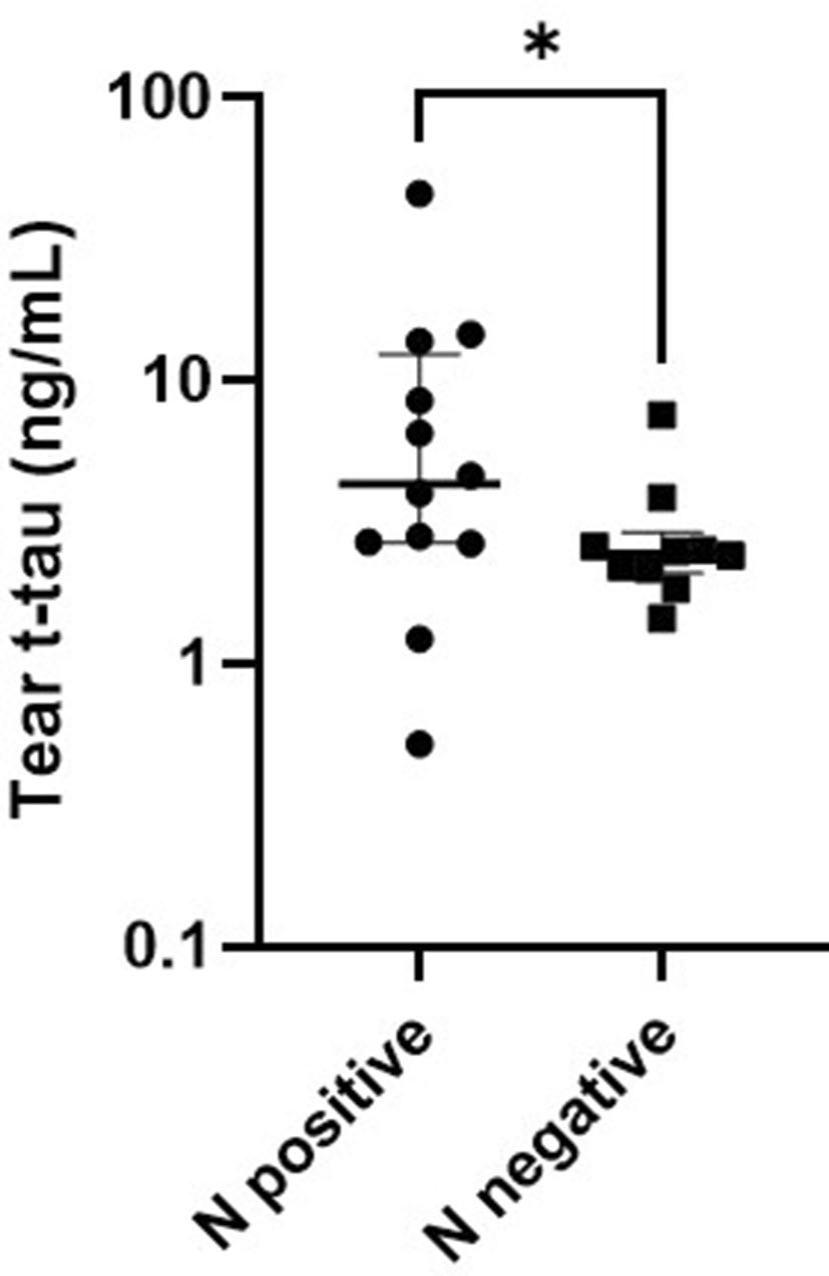
T-tau biomarker levels across ATN groups. Tear t-tau levels in patients with neurodegeneration (N positive) compared to patients without neurodegeneration (N negative). Bars represent median and IQR. P-values were determined using Kruskal–Wallis testing including Bonferroni correction. *p < 0.05.

Patients in the CSF group were subdivided along the A/T/N classification. Tear t-tau levels were elevated in patients with neurodegeneration (N positive) (median 4.30, IQR 9.76) compared to patients without neurodegeneration (N negative) (median 2.54, IQR 0.83) (p = 0.04) (Fig. 2). The number of samples with detectable levels of tear AB42 and tear p-tau was too low to allow comparison within A and T groups.

#### Correlation of biomarkers in tears and CSF

**Figure 3:**
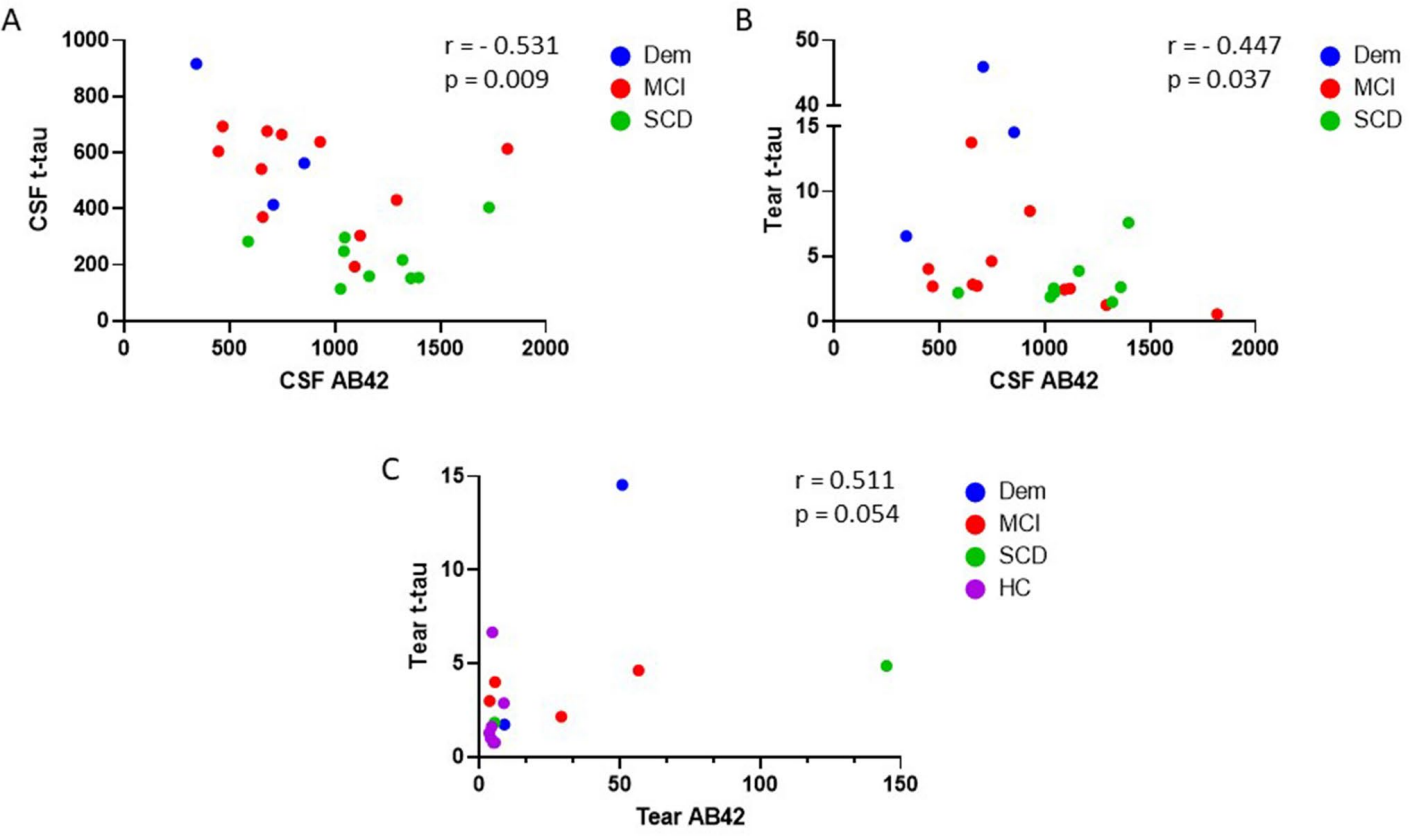
Correlation matrix between t-tau and AB42. Correlation between t-tau and AB42 for **(A)** CSF, **(B)** tear and CSF and **(C)** tear. Results are presented as Spearman rank correlation coefficients (r) and p-values (p).

Table 4 presents the correlation matrix between biomarkers in tears (of the total study group) and between tears and CSF (of the CSF group). All AB species in tear fluid were positively correlated with each other (all r > 0.874, p < 0.01). In CSF, there were significant correlations between AB42 and t-tau, AB42 and p-tau and, t-tau and p-tau. We found negative correlations between CSF AB42 and tear AB40 and between CSF AB42 and tear t-tau. Figure 3 illustrates the correlations between AB42 and t-tau for each body fluid.

**Table 4:** Correlations of biomarkers within the total study population (left) and the CSF group (right).

	Total group (n = 65)					CSF group (n = 23)		
	Tear AB38	Tear AB40	Tear AB42	Tear t-tau	Tear p-tau- 231	CSF AB42	CSF t-tau	CSF p-tau-181
Tear AB38	1.000	0.874^c^	0.964^b^	0.552	–	**–**	**–**	**–**
Tear AB40		1.000	0.943^c^	0.072	0.261	−0.533^b^	0.240	0.213
Tear AB42			1.000	0.511	–	**–**	**–**	**–**
Tear t-tau				1.000	0.056	−0.447^a^	0.328	0.332
Tear p-tau-231					1.000	**–**	**–**	**–**
CSF AB42						1.000	−0.531^c^	−0.475^a^
CSF t-tau							1.000	0.967^c^
CSF p-tau-181								1.000

Data are presented as Spearman rank correlation coefficient (r). Correlations with n < 10 pairs were omitted.

*AB* amyloid beta, *CSF* cerebrospinal fluid, *t-tau* total tau, *p-tau-231* tau phosphorylated at threonine 231, *p-tau-181* tau phosphorylated at threonine 181.

^a^p < 0.05, ^b^p < 0.01, ^c^p < 0.001.

## Discussion

This study investigated tear levels of amyloid-beta peptides and tau protein in patients with cognitive impairment and healthy controls. While AB40 and total-tau were well detectable, AB38, AB42 and p-tau were detectable in less than half of the collected tear fluid samples. These results are comparable to CSF and blood samples where AB40 has been found the most abundant soluble AB peptide species^22,23^. Similarly, t-tau levels in CSF are five to ten times higher than p-tau levels. Levels of p-tau were undetectable in our tear fluid samples from healthy controls. This is an interesting finding since the determination of p-tau in CSF may increase the specificity and sensitivity in the detection of AD as opposed to t-tau^12^. In addition, tear levels of AB42 were much more detectable in healthy controls compared to patients. This finding also seems to reflect the physiological condition. However, tests with higher sensitivity for p-tau and AB42 are needed to confirm these observations in tear fluid, and to allow investigation of the AB42/AB40 ratio in tear fluid. Unfortunately, tear fluid, in contrast to CSF and blood, requires sample preprocessing that involves dilution of the original sample volume. Moreover, ultrasensitive immunoassays often require large sample volumes and are limited available as multiplex. Working with biological samples with limited sample volumes such as tear fluid is therefore challenging.

Previous studies investigating tear fluid samples of AD patients used mass spectrometry and were therefore not able to detect amyloid-beta and tau, since (untargeted) mass spectrometry is less sensitive for low abundant proteins than immunoassays^24,25^. Kallo et al*.* used mass spectrometry to compare ten priorly selected abundant proteins in tear fluid samples from AD patients versus controls and observed differences for lipocalin-1, lactotransferrin, extracellular glycoprotein lacritin, lysozyme-C, prolactin inducible protein and dermcidin^24^. Kenny et al. used mass spectrometry on tear fluid from AD patients versus controls^25^. They identified a panel of 12 proteins that were differentially expressed in tear fluid of AD patients compared to controls. Among these, elongation initiation factor 4E (eIF4E, involved in protein synthesis) was only detectable in AD samples and undetectable in samples from controls.

We found elevated levels of tear AB40 and tear t-tau in patients with cognitive impairment, and those levels increased with increasing disease severity. However, the majority of differences were not statistically significant, most probably due to large sample variability and small sample size. The level of tear t-tau was significantly higher in dementia patients compared to SCD patients in the CSF group, in the same way as CSF t-tau. Interestingly, all these dementia patients displayed CSF t-tau levels above the diagnostic threshold, while this was not the case for all SCD patients, except for one patient. In view of this, we used the A/T/N classification system of AD biomarkers that divides patients into binary classes^3,26^. “N” refers to a quantitative or topographic biomarker of neurodegeneration or neuronal injury (CSF t-tau). The + /− categorization is based on lab-specific threshold values. Tear t-tau levels were elevated in N positive patients compared to N negative patients. These results suggest that tear fluid may mirror CSF with regards to (t-)tau pathology. Cutoff values for tear fluid are however yet to be established and require further investigation.

Our study is in line with others efforts that identified tear biomarkers for different neurological conditions, such as TNF-alpha^27^ and oligomeric alpha-synuclein^28^ for Parkinson’s disease and alpha-1 anti-chymotrypsin for multiple sclerosis^29^. In those studies, the levels of TNF-alpha and alpha-1 antichymotrypsin were also elevated in the CSF of parkinsonian patients and multiple sclerosis patients, respectively. Together, these studies support the hypothesis that tear fluid may predispose to mirror pathophysiological changes in the central nervous system.

In our investigation, we were able to correlate biomarkers levels in tear fluid with CSF in a subset of patients from whom CSF biomarker levels were available from clinical diagnostic purposes. We observed that CSF AB42 and tear t-tau were negatively correlated in the same way as CSF AB42 and CSF t-tau. On the other hand, AB42 and t-tau were positively correlated in tear fluid, similarly to what has been found in plasma^30^ The inter-correlation between the three AB species in tear fluid resembles both CSF^22^ as well as plasma^30^, while there was a lack of correlation for t-tau levels between tear fluid and CSF. The latter finding is in agreement with plasma tau^31^ and together these results suggest that tear fluid seems to resemble better with peripheral fluids, such as plasma, compared to CSF. A larger study comparing those three body fluids for a wide range of biomarkers would clarify this.

Limitations of our study are the small sample size, especially in the dementia group, and the limited availability of information on the etiology of the disease. This is because our study subjects were recruited from a pool of patients who visited the memory clinic because of cognitive complaints. In most cases, their clinical diagnosis is not accompanied with CSF sampling or PET imaging as these are not routinely performed in regular care settings. Although we believe that the current study population is more clinically relevant than comparing severely affected patients with healthy controls (as it are patients with early symptoms that would benefit from future tear-based biomarker tests), larger sample size studies are warranted to confirm and validate our results.

## Methods

### Participants

A total of 56 patients and nine cognitively healthy controls (HC) were enrolled in this study. Patients were recruited from the BioBank Alzheimer Center Limburg (BB-ACL), Maastricht, the Netherlands. Inclusion criteria were an MMSE score ≥ 20 and a CDR score from 0 to 1, thereby including patients across the whole clinical spectrum (i.e. from subjective cognitive disorder to (mild) dementia). Exclusion criteria at baseline were neurological diseases (such as Normal Pressure Hydrocephalus, Morbus Huntington, brain tumor, epilepsy, encephalitis, recent Transient Ischemic Attack (TIA) or cerebrovascular accident (CVA) (< 2 years), or TIA/CVA with concurrent (within three months) cognitive decline) and a history of psychiatric disorders (such as schizophrenia, bipolar disorder or psychotic problems, current major depressive disorder (within 12 months), or alcohol abuse). Healthy controls were recruited at the University Eye Clinic Maastricht, the Netherlands. The medical ethics committee of the Maastricht University Medical Center, the Netherlands approved the study protocol and this study followed the tenets of the current version of the Declaration of Helsinki. Written informed consent was obtained from all participants.

### Clinical diagnosis and assessment

During their visit to the memory clinic, all patients underwent standardized clinical assessments including neuropsychological assessments, blood tests, MRI cerebrum and, from a subset of patients, CSF sampling. A diagnosis of dementia was made according to regular DSM-5 criteria for neurocognitive disorders^32^. A diagnosis of mild cognitive impairment (MCI) was based on the clinical National Institute on Ageing-Alzheimer’s Association (NIA-AA) criteria, which entails that patients have impaired cognitive functioning but sufficiently preserved function performance such that a dementia diagnosis cannot be made^33^. All other patients were classified as subjective cognitive decline (SCD), indicating the presence of a cognitive complaint initiation the visit to the memory clinic, but having no impairment in cognitive test performances and activities of daily living. The global cognitive status was evaluated using the Mini-Mental State Examination (MMSE)^34^.

### CSF collection and biochemical analyses

CSF was collected via a lumbar puncture. After collection, CSF was centrifuged at room temperature, aliquoted and stored at −80 °C until analysis. The levels of amyloid-beta-42 (AB42), total tau (t-tau) and phosphorylated at threonine 181 tau (p-tau) were measured using commercially available single-parameter ELISA methods (respectively Innotest® beta-amyloid (1–42) and Innotest® hTAU-Ag; Innogenetics, Ghent, Belgium) at the department of Neurochemistry, Radboud University MC, Nijmegen the Netherlands. Diagnostic cutoff values were 500 pg/mL for AB42, 350 pg/mL for t-tau and 85 pg/mL for p-tau.

### A/T/N/ classification

We used the A/T/N classification system of AD biomarkers that divides patients into 3 binary classes^3,26^. “A” refers to the value of an AB biomarker (CSF AB42); “T,” the value of a tau pathology biomarker (CSF p-tau); and “N,” a quantitative or topographic biomarker of neurodegeneration or neuronal injury (CSF t-tau). The + /− categorization is based on threshold values.

### Tear fluid collection and biochemical analyses

Tear fluid was collected from the left and right eye via Schirmer’s tear strips (TEAR strips, Contacare Ophthalmics and diagnostics, Gujarat, India) without topical anesthesia. Tear fluid was extracted from each Schirmer’s strip by agitating small cut pieces of these strips in 60 µL PBS, Tween20 0.5% and cOmplete™ Protease Inhibitor Cocktail (Roche, Basel, Switzerland) at 4 °C for 1.5 h^35,36^. Tear fluid was then eluted by centrifugation and stored at −80 °C until further use. Levels of amyloid-beta peptides (AB38, AB40 and AB42), t-tau and p-tau in eluted tear fluid were determined using the Human Aβ triplex Ultra-Sensitive assay and the Phospho(Thr231)/Total Tau duplex assay (Meso Scale Discovery, Rockville, MD, USA) at the department of Internal Medicine, School for Cardiovascular Diseases (CARIM), Maastricht University Medical Center, Maastricht, the Netherlands. The absolute concentrations were then normalized to the wetting length (tear fluid volume) and dilution (extraction buffer volume) as described elsewhere^35,37^. Values below the lower limit of detection were excluded.

### Statistical analysis

Statistical analyses were performed using GraphPad Prism version 9.0.0 (GraphPad Software, San Diego, CA, USA). Data in tables are presented as medians with interquartile range (IQR). Differences in continuous variables across diagnostic groups were analyzed using Kruskal–Wallis testing with multiple comparison. Differences in continuous variables across A/T/N groups were examined by the Mann–Whitney U test. Differences in categorical variables were analyzed using the Fisher exact test. Correlations were assessed using Spearman Rank correlation analysis. Correlations with less than 10 pairs were omitted.

## Abbreviations

AD: Alzheimer’s disease
HC: Healthy controls
SCD: Subjective cognitive disorder
MCI: Mild cognitive impairment
Dem: Dementia
CDR: Clinical dementia rating
MMSE: Mini-Mental State Examination
CSF: Cerebrospinal fluid
AB42: Amyloid-beta 42
IQR: Interquartile range

## References

[1] Hardy JA, Higgins GA (1992). Alzheimer's disease: The amyloid cascade hypothesis. Science.

[2] Shoghi-Jadid K (2002). Localization of neurofibrillary tangles and beta-amyloid plaques in the brains of living patients with Alzheimer disease. Am. J. Geriatric Psychiatry.

[3] Jack CR (2018). NIA-AA research framework: Toward a biological definition of Alzheimer's disease. Alzheimers Dement..

[4] Koronyo-Hamaoui M (2011). Identification of amyloid plaques in retinas from Alzheimer's patients and noninvasive in vivo optical imaging of retinal plaques in a mouse model. Neuroimage.

[5] den Haan J (2018). Amyloid-beta and phosphorylated tau in post-mortem Alzheimer's disease retinas. Acta Neuropathol. Commun..

[6] van de Kreeke JA (2020). Ocular biomarkers for cognitive impairment in nonagenarians; A prospective cross-sectional study. BMC Geriatr..

[7] Javaid FZ, Brenton J, Guo L, Cordeiro MF (2016). Visual and ocular manifestations of Alzheimer's disease and their use as biomarkers for diagnosis and progression. Front. Neurol..

[8] Lim JK (2016). The eye as a biomarker for Alzheimer's disease. Front. Neurosci..

[9] Armstrong RA (2009). Alzheimer's disease and the eye☆. J. Optometry.

[10] Alber J (2020). Developing retinal biomarkers for the earliest stages of Alzheimer's disease: What we know, what we don't, and how to move forward. Alzheimers Dement..

[11] Blennow K (2004). Cerebrospinal fluid protein biomarkers for Alzheimer's disease. NeuroRx.

[12] Blennow K, Hampel H, Weiner M, Zetterberg H (2010). Cerebrospinal fluid and plasma biomarkers in Alzheimer disease. Nat. Rev. Neurol..

[13] Cohen AD (2019). Fluid and PET biomarkers for amyloid pathology in Alzheimer's disease. Mol. Cell. Neurosci..

[14] Olsson B (2016). CSF and blood biomarkers for the diagnosis of Alzheimer's disease: A systematic review and meta-analysis. Lancet Neurol..

[15] Bălașa AF, Chircov C, Grumezescu AM (2020). Body fluid biomarkers for Alzheimer's disease-an up-to-date overview. Biomedicines..

[16] Lee JC, Kim SJ, Hong S, Kim Y (2019). Diagnosis of Alzheimer’s disease utilizing amyloid and tau as fluid biomarkers. Exp. Mol. Med..

[17] Zetterberg H, Burnham SC (2019). Blood-based molecular biomarkers for Alzheimer’s disease. Mol. Brain.

[18] Cappelli F (2020). Evaluating the correlation between Alzheimer's amyloid-β peptides and glaucoma in human aqueous humor. Transl. Vis. Sci. Technol..

[19] Wright LM (2019). Association of cognitive function with amyloid-beta and tau proteins in the vitreous humor. J. Alzheimers Dis..

[20] Subramanian ML (2020). Neurofilament light chain in the vitreous humor of the eye. Alzheimer's Res. Ther..

[21] Van Setten GB (1996). Beta-amyloid protein protein precursor expression in lacrimal glands and tear fluid. Invest. Ophthalmol. Vis. Sci..

[22] Schoonenboom NS (2005). Amyloid beta 38, 40, and 42 species in cerebrospinal fluid: More of the same?. Ann. Neurol..

[23] Hilal S (2018). Plasma amyloid-beta levels, cerebral atrophy and risk of dementia: A population-based study. Alzheimers Res. Ther..

[24] Kallo G (2016). Changes in the chemical barrier composition of tears in Alzheimer's disease reveal potential tear diagnostic biomarkers. PLoS ONE.

[25] Kenny A (2019). Proteins and microRNAs are differentially expressed in tear fluid from patients with Alzheimer's disease. Sci. Rep..

[26] Jack CR (2016). A/T/N: An unbiased descriptive classification scheme for Alzheimer disease biomarkers. Neurology.

[27] Comoglu SS, Guven H, Acar M, Ozturk G, Kocer B (2013). Tear levels of tumor necrosis factor-alpha in patients with Parkinson's disease. Neurosci. Lett..

[28] Hamm-Alvarez SF (2019). Oligomeric α-synuclein is increased in basal tears of Parkinson’s patients. Biomark. Med..

[29] Salvisberg C (2014). Exploring the human tear fluid: Discovery of new biomarkers in multiple sclerosis. Proteom. Clin. Appl..

[30] Verberk IM (2018). Plasma amyloid as prescreener for the earliest A lzheimer pathological changes. Ann. Neurol..

[31] Zetterberg H (2013). Plasma tau levels in Alzheimer's disease. Alzheimer's Res. Ther..

[32] Sachdev PS (2014). Classifying neurocognitive disorders: The DSM-5 approach. Nat. Rev. Neurol..

[33] Albert MS (2011). The diagnosis of mild cognitive impairment due to Alzheimer's disease: Recommendations from the National Institute on Aging-Alzheimer's Association workgroups on diagnostic guidelines for Alzheimer's disease. Alzheimers Dement..

[34] Folstein MF, Folstein SE, McHugh PR (1975). “Mini-mental state”: A practical method for grading the cognitive state of patients for the clinician. J. Psychiatr. Res..

[35] Khamar P (2019). Dysregulated tear fluid nociception-associated factors, corneal dendritic cell density, and vitamin D levels in evaporative dry eye. Invest. Ophthalmol. Vis. Sci..

[36] Sethu S (2016). Correlation between tear fluid and serum vitamin D levels. Eye Vis. (Lond).

[37] Vinekar A (2021). Tear fluid angiogenic factors: Potential noninvasive biomarkers for retinopathy of prematurity screening in preterm infants. Invest. Ophthalmol. Vis. Sci..

